# Key molecular DNA damage responses of human cells to radiation

**DOI:** 10.3389/fcell.2024.1422520

**Published:** 2024-07-10

**Authors:** Chencheng Zhang, Jibin Liu, Jun Wu, Kamakshi Ranjan, Xiaopeng Cui, Xingdan Wang, Dianzheng Zhang, Shudong Zhu

**Affiliations:** ^1^ Cancer Research Center, Nantong Tumor Hospital, Nantong, China; ^2^ Cancer Research Institute, The Affiliated Tumor Hospital of Nantong University, Nantong, China; ^3^ Cancer Research Center, Nantong, China; ^4^ Nantong Tumor Hospital, Nantong, China; ^5^ Department of Bio-Medical Sciences, Philadelphia College of Osteopathic Medicine, Philadelphia, PA, United States; ^6^ Department of General Surgery, The Affiliated Hospital of Nantong University, Nantong, China; ^7^ Department of Radiotherapy, Nantong Tumor Hospital, The Affiliated Tumor Hospital of Nantong University, Nantong, China; ^8^ Argus Pharmaceuticals, Changsha, China

**Keywords:** radiation, DNA damage, repair, apoptosis, ATM, ATR, DNA-PK, cell cycle arrest

## Abstract

Our understanding of the DNA damage responses of human cells to radiation has increased remarkably over the recent years although some notable signaling events remain to be discovered. Here we provide a brief account of the key molecular events of the responses to reflect the current understanding of the key underlying mechanisms involved.

## Introduction

When human cells are under ionizing radiation (IR), either in radiation therapy or in adverse environmental conditions, DNA damage occurs, including single-strand breaks (SSBs), base pair damage, and the most lethal double-strand breaks (DSBs); various signaling cascades are initiated centering around DNA-dependent protein kinase (DNA-PK), ATM, and ATR, forming an interconnected network. However, many signaling events are yet to be identified, and new perspectives are emerging. This review aims to providing a comprehensive yet brief account of the key molecular events of the radiation responses to reflect the current understanding of the key underlying mechanisms involved.

## Radiation-induced cellular damages

IR is prevalent in various aspects of daily life, causing cellular damage through multiple pathways. The derived radiotherapy utilizes high-energy radiation from specialized equipment to irradiate cancerous cells, to inhibit their growth, reproduction, and spread, serving as one of the primary means for treating malignant tumors. Since cells and tissues are composed of about 80% of water, a lot of the radiation damage is induced via the radiolysis of water, leading to the generation of reactive oxygen species (ROS) and reactive nitrogen species (RNS) free radicals (Vorotnikova et al., 2010). These free radicals cause mitochondrial dysfunction and oxidative stress by affecting multiple enzymes such as nicotinamide adenine dinucleotide phosphate oxidase (NADPH oxidase), lipoxygenase (LOX), nitric oxide synthase (NOS), and cyclooxygenase (COXs). These radicals and oxidative stress molecules lead to either direct or indirect oxidative DNA damage, resulting in various cellular survival regulatory mechanisms such as mitotic catastrophe, senescence, apoptosis, and autophagy (Wei et al., 2019).

In antitumor therapy, IR not only induces stress-induced regulatory cell death but also drives antitumor immune responses by affecting tumor-associated cytokines or specific antigens, thereby inducing immunogenic cell death (Zhu et al., 2021). In endothelial cells and the hematopoietic system, IR and ROS disrupt cell membrane integrity, causing localized calcium influx, lysosomal fusion, and inducing cell death through biophysical mechanisms (Ferranti et al., 2020). Radiation can also cleave disulfide bonds and alter protein conformations, disrupting normal biological functions of proteins and affecting cellular activities (Fitzner et al., 2023). On DNA, IR induces the generation of radical cations (holes), leading to DNA-protein crosslinks (DPCs) (Wen et al., 2023). Additionally, IR causes significant amounts of single-strand and DSBs through the rapid decay of transient molecular resonances localized on fundamental DNA components (Boudaïffa et al., 2000).

## DNA damage induced by IR

DNA damage induced by IR includes both physical and chemical damage. Physical damage occurs when ionized particles directly interact with DNA through energy deposition of ionization and excitation, while chemical damage results from indirect interactions due to free radical reactions produced by high-energy particles in the large amount of water surrounding DNA (Nikjoo et al., 2001; Matsuya et al., 2020). IR-induced DNA damages includes base damage (BD), apurinic/apyrimidinic (AP) sites, SSBs, DSBs, and DPCs (Nakano et al., 2022).

Regarding DNA physical damage, high-energy charged particles in radiation (i.e., fast electrons, protons, or ions) or primary high-energy photons generate primary ionization and excitation as they propagate through biological tissues. Most of the energy from these fast particles is deposited into a large number of cations and secondary electrons (SEs) (Gao et al., 2021). SEs, which are considered low-energy electrons (LEEs) below 20 eV, carry most of the energy deposited by high-energy radiation in cells. These SEs are a significant cause of various types of DNA damage induced by irradiation (Kumar et al., 2024). SEs can produce further ionization and transient anions (TAs), where the separation of additional electrons can put molecules or molecular subunits in a vibrational or electronic excited state, leading to the production of high-energy ions and radicals (Gao et al., 2021). Initially, electrons are captured by bases on DNA, forming nuclear-excited TAs. The electron then separates from the base anion, leaving the base in a dissociated state. The base is damaged, and the separated electron transfers to another site for dissociative electron attachment (DEA). Depending on the attachment site of the transferred electron (i.e., base or phosphate group), three different types of local cluster damage can be induced. Transfer to the opposite base will result in damage to two adjacent bases (BD), while transfer to the same or opposite strand’s phosphate units can lead to strand breaks through C-O bond cleavage (SSB, DEA + BD) or DSB (Dong et al., 2021; Gao et al., 2021). Electron hopping between bases can cause BD or SSB at locations further from the initial electron capture site.

In terms of DNA chemical damage, IR produces many radicals through the radiation of water, including •OH radicals, single-electron oxidants, singlet oxygen (1O2), and hypochlorous acid (HOCl), which can oxidize and generate single base lesions. The most common oxidation reaction occurs on guanine due to its low redox potential, resulting in 8-oxo-7, 8-dihydroguanine (8-oxoG) and 2, 6-diamino-4-hydroxy-5-formamidopyrimidine (FapyG), collectively known as oxidative base damage (Cadet and Wagner, 2013). Additionally, the oxidative action of free radicals on DNA and proteins leads to the production of DPCs. Actin, histones, and other proteins have been identified as crosslinked proteins, with the covalent bonds between DNA and proteins including thymine-lysine and guanine-lysine esters (Nakano et al., 2017).

Clustered DNA damage, the main harmful effect of IR on cells, is classified according to the complexity of local damage (within 10–15 base pairs or one to two helical turns of DNA) into simple clustered DNA damage (damage complexity = 2) and complex clustered DNA damage (damage complexity ≥3) (Danforth et al., 2022; Nakano et al., 2022). Simple clustered DNA damage includes simple base damage clusters (BDCs) containing two adjacent bases and/or AP damaged and simple DSBs without associated base/AP damage near the DSB ends. Complex clustered DNA damage includes complex BDCs containing three or more adjacent bases and/or AP damaged and complex DSBs containing one or more bases and/or AP damage near the DSB ends (Nakano et al., 2022).

By studying the structural simulation of radiation tracks, Nikjoo and colleagues combined the location of DNA damage sites within the local area of IR, the complexity of damage, and the proportion of BD, dividing DNA damage into 10 categories across three tiers. The first tier consists of the most basic SSB (a single break in the phosphodiester bond on a single DNA strand) and DSB (two SSBs located on opposite DNA strands with a maximum separation distance of less than 10 bp) (Schipler and Iliakis, 2013; Mladenova et al., 2022). The second tier contains four categories: SSB+ (two or more SSBs on the same strand), 2SSB (two SSBs on opposite strands but not constituting a DSB), DSB+ (a combination of one DSB and one or more SSBs), and DSB++ (two or more DSBs in the local area). The third tier encompasses four types: complex SSB (SSBc), defined as the combination of SSB+ and 2SSB observed in the local region of IR; and complex DSB (DSBc), defined as a combination of DSB+ and DSB++. In cases where the local region contains at least one BD in the SSBc, it is referred to as SSBcd, whereas an SSBc containing one or more BDs is termed DSBcd (Nikjoo et al., 1999).

## Key ATM signaling events under irradiation

**FIGURE 1:**
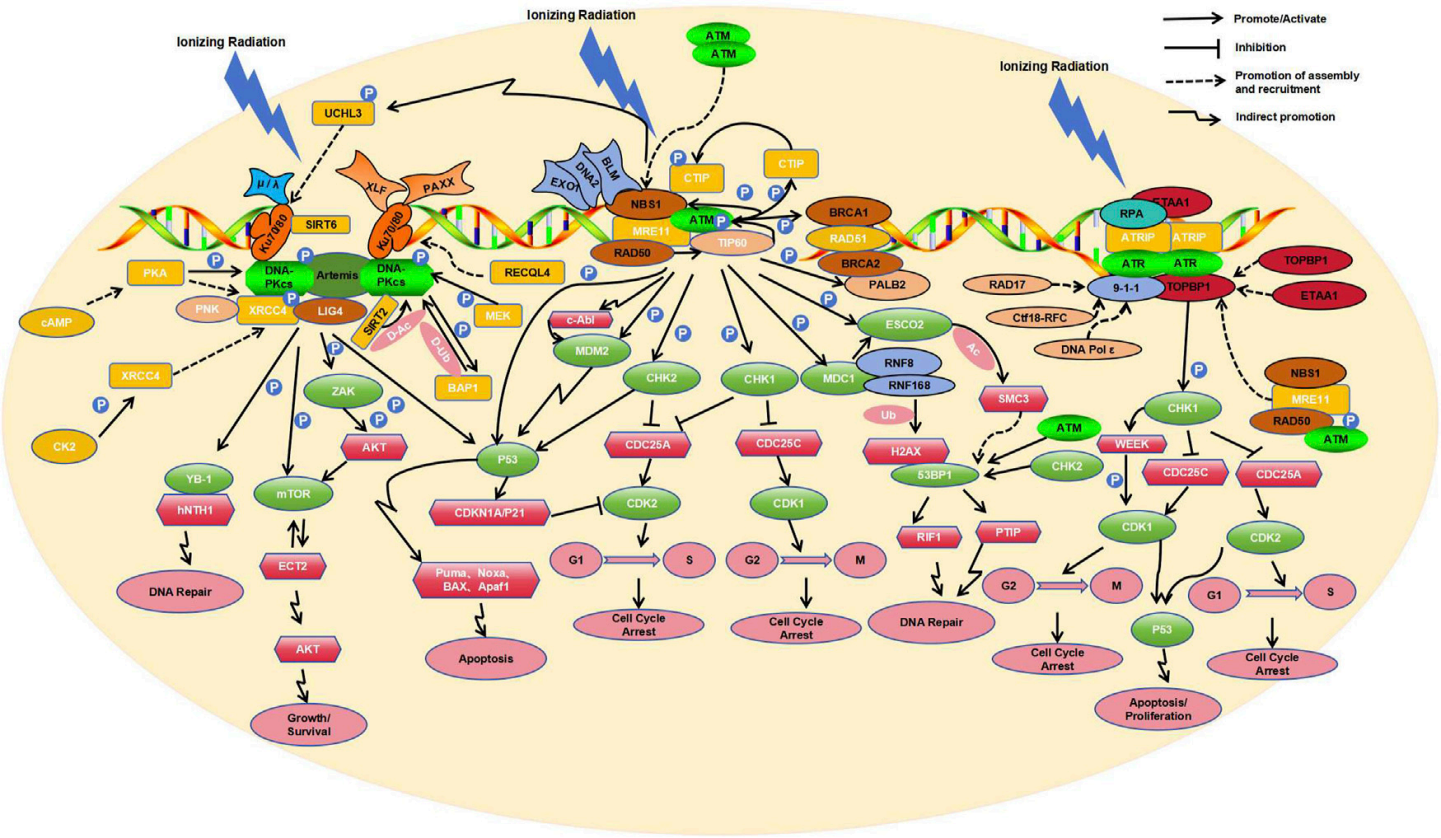
Key molecular responses of human cells to radiation.

The molecular responses of human cells to radiation-induced DNA damage are controlled by three related kinases including ATM, ATR, and DNA-PK (Blackford et al., 2017; Figure 1). ATM participates in the homologous recombination (HR) repair process (Bakr et al., 2015), mainly repairing DSBs in the S and G2 phases of the cell cycle (Ouyang et al., 2021). This process is initiated by ionizing irradiation induced DSBs. Next, the MRN protein complex, composed of MRE11, RAD50, and NBS1, identifies the termini of the damaged DNA and is rapidly recruited to the DSB sites. In the MRN complex, RAD50 helps to anchor MRN to the DSB sites (Zabolotnaya et al., 2020), MRE11 endonucleolytically processes the break ends (Gut et al., 2022), and NBS1, acting as a substrate of ATM, facilitates the recruitment of ATM (Abdisalaam et al., 2022). Following recruitment by the MRN complex, dimeric inactive ATM dissociates into the monomeric active form after auto-phosphorylation at sites S1981, S367, and S1893 (counter-acted by PP2A). The active ATM then orchestrates complex downstream phosphorylation cascades (Goodarzi et al., 2004; Kalev et al., 2012).

Tip60, a histone acetyltransferase (HAT), is also able to sense DNA damage via its chromodomain, which interacts with the methylated histones exposed at the DNA damage site. This interaction activates the HAT activity of Tip60 independent of ATM activity. Tip60 then acetylates ATM at its lysine residues, activating ATM to initiate downstream signaling (Sun et al., 2005; Sun et al., 2009). Active ATM also reciprocally acts on each component of the MRN complex, i.e., ATM modulates downstream signal transduction through phosphorylation of RAD50 (Gatei et al., 2011), enhances nuclease activity of MER11 via phosphorylation (Kijas et al., 2015), and influences the S phase checkpoint via phosphorylation of NBS1 (Lim et al., 2000). Additionally, activated ATM can phosphorylate the T859 site of CtIP (C-terminal-binding protein interacting protein) (Wang et al., 2021), which allows CtIP to bind NBS1 in the MRN complex, thereby enhancing the endonuclease activity of MRE11 in MRN (Zdravković et al., 2021). Furthermore, HR nucleases EXO1 and DNA2 are likely recruited by MRN in a similar manner, along with the repair helicase BLM, albeit at a relatively longer range from the DSBs compared to MRE11 (Whelan and Rothenberg, 2021).

Moreover, BRCA1 can sense DSBs and recruit BRCA2 and RAD51 through its interaction with PALB2 (Bouwman et al., 2010; Evans and Longo, 2014). In this process, activated ATM plays the role in phosphorylating PALB2, allowing it to co-localize with its partner BRCA2 at the DNA damage site (Simhadri S et al., 2019). After the nucleotide excision, RAD51 is loaded onto the resultant 3′overhangs (Muhammad et al., 2023). BRCA2 facilitates loading of RAD51 onto replication protein A (RPA)-coated single-stranded DNA (ssDNA) and prevents RAD51 dissociation from ssDNA (Jiang et al., 2021). RAD51 loaded onto ssDNA forms a nucleoprotein filament (Wei et al., 2019) that allows invasion into homologous DNA duplexes, which HR then uses as templates for DNA synthesis and repair (Tan et al., 2020).

In addition to phosphorylating and recruiting the aforementioned components to DSBs, activated ATM also phosphorylates various other substrates, including PD-L1 (Permata et al., 2021), CHK2 (Mustofa et al., 2020), FANCD2 (Ishiai, 2021), NF-κB (Hadian and Krappmann, 2011), H2AX (Arnould et al., 2021), and p53 (Roos et al., 2013). Among these molecular targets, CHK2 plays a key role in the checkpoint control (Smith et al., 2020). ATM phosphorylates CHK2 at residue T68 (Zhang et al., 2022), leading to dimerization of the kinase domain of CHK2 and enabling its full enzymatic activity (Cai et al., 2009). Activated CHK2 subsequently phosphorylates Cdc25A at S124 (Wei et al., 2015) and Cdc25C at S216 (Wei et al., 2010), leading to their inactivation/degradation. The inactivation/degradation of Cdc25A blocks its dephosphorylation of cyclin-dependent kinase2 (CDK2) on Y15 (Ditano et al., 2021), inhibiting the cell cycle transition from G1 to S phase (Liu et al., 2023). Similarly, the inactivation/degradation of Cdc25C blocks its dephosphorylation of CDK1, inhibiting the cell cycle transition from G2 to M phase (Huang R et al., 2020). Both ATM and CHK2 can phosphorylate p53 at residues S15 and S20, leading to its activation (Canman et al., 1998; Choi et al., 2012). Activated p53 promotes the transcription of CDKN1A, encoding the cyclin-dependent kinase inhibitor p21 (Ben-Oz et al., 2023), which also inhibits CDK2 activity (Yamada et al., 2016). Notably, ATM has been shown to bind p53 mRNA, suggesting a potential novel feedback regulatory mechanism at the level of p53 mRNA (Karakostis et al., 2024). Therefore, ATM-dependent phosphorylation of p53 and CHK2 contributes to cell cycle arrest and DNA repair (Usluer et al., 2024). On the other hand, p53 activation also induces the transcription of pro-apoptotic factors, including Puma, Noxa, BAX, and Apaf1, which promote apoptosis under prolonged damage (Bieging et al., 2014), indicating a balanced mechanism of repair or cell elimination to preserve cell integrity.

ATM can also phosphorylate MDC1, which then recruits the E3 ubiquitin ligases RNF8/RNF168 to ubiquitinate histone H2AX, thereby facilitating the binding of 53BP1 (Lee et al., 2017). Activated ATM is capable of phosphorylating the acetyltransferase ESCO2 at serine 196 and threonine 233 residues (Fu et al., 2023). MDC1 recognizes the phosphorylated ESCO2 and recruits it to the DSB site. ESCO2-mediated acetylation of SMC3 regulates chromatin structure at the site of DSBs, which is essential for the recruitment of 53BP1 and the formation of 53BP1 microdomains (Fu et al., 2023). 53BP1 can be phosphorylated by ATM and CHK2, recruiting RIF1 (Rap1-Interacting Factor 1) and PTIP (Pax Transactivation Domain Interacting Protein) to the DSB. This protects the DNA break ends from resection and competes with BRCA1-HR repair during the G1 phase, promoting the occurrence of Non-Homologous End Joining (NHEJ) (Feng et al., 2015), which is supplementary to the HR.

It’s noteworthy that activated ATM induces phosphorylation of BRCA1 at S988 during S phase and at S1423 during G2/M phase, showing differential effects on BRCA1 localization and function depending on the phosphorylation site (Okada and Ouchi, 2003). Phosphorylated BRCA1 also interacts with ATR (Bunch et al., 2021) to regulate p53-related DNA damage repair (Kim et al., 2009). By phosphorylating the histone variant H2AX at S139, ATM facilitates the recruitment of damage signaling factors to initiate DNA repair (Burma S et al., 2001). ATM-triggered TAK1-dependent phosphorylation of JNK can activate AP-1 and transcription of the cellular prion protein PrPC. The ATM-TAK1-PrPC axis is implicated in the development of tumor cell resistance to radiation therapy (Bernardino-Sgherri et al., 2021). Additionally, ATM can modulate the cytoskeletal protein intermediate filament synemin in DSB repair and radiation resistance through the c-Abl tyrosine kinase (Deville et al., 2020). ATM and c-Abl also regulate DNA damage repair by phosphorylating the E3 ligase Mdm2 (Correction to Supporting Information for Chibaya et al., 2021).

## Key DNA-PK signaling events under irradiation

DNA-PK primarily orchestrates the NHEJ pathway in cellular DSB repair (Head et al., 2023), with assistance from the Ku70/Ku80 heterodimer (Gell and Jackson, 1999). Through their shared topological structures, Ku70 and Ku80 form a ring structure that detects DSBs and encircles the double-stranded DNA (Walker et al., 2001). The heterodimer then recruits the catalytic subunit of DNA-dependent protein kinase (DNA-PKcs) via Ku80’s C-terminal region which forms a flexible Arm to recruits DNA-PKcs, causing its autophosphorylation at S2056, S3950, and T2609, and kinase activation, thereby activating DNA-PK (Hammel et al., 2010). On the other hand, upon phosphorylation by CK2 at T233, XRCC4 physically links polynucleotide kinase (PNK) with DNA ligase IV (LIG4) to form the PNK-XRCC4-LIG4 complex for both the end processing and ligation at DSBs in DNA-PK-mediated NHEJ (Koch et al., 2004). Additionally, both XLF (XRCC4 paralog XRCC4-Like Factor) and PAXX (XRCC4 and XLF paralog) interact with the Ku70/Ku80 heterodimer through the Ku-binding motif (KBM) to stabilize NHEJ complexes on DNA (Wang et al., 2018). When complexed with DNA-PKcs, Artemis is phosphorylated and converted from a nucleic acid exonuclease to a nucleic acid endonuclease (Ma et al., 2002). Artemis also recruits NHEJ-related factors such as DNA polymerases μ and λ to restore DNA integrity (Mahaney et al., 2009; Anisenko et al., 2021). These polymerases interact with Ku through their BRCT structure domain (Zhao et al., 2020), promoting end processing and DNA hairpin opening (Sonmez et al., 2024).

Phosphorylation and auto-phosphorylation play pivotal roles in the activation of DNA-PKcs. Cyclic adenosine monophosphate (cAMP) modulates the phosphorylation of DNA-PKcs at S2056 and T2609 through protein kinase A (PKA), in a cell type-specific manner (Noh and Juhnn, 2020a); MEK5 kinase phosphorylates DNA-PKcs at S2056 site, which is implicated in NHEJ (Broustas et al., 2020). Activated DNA-PK phosphorylates BRCA1-associated protein 1 (BAP1) at S395, and BAP1, in turn, deubiquitinates DNA-PKcs, establishing a positive feedback loop between the two (Sato et al., 2024). During the assembly of DNA-PK complexes and accessory factors at DSBs, SIRT2 deacetylates DNA-PKcs, promoting its binding to Ku (Correction to Supporting Information for Chibaya et al., 2021). Conversely, the deacetylase SIRT6 directly binds to Ku80 and enhances the interaction between Ku80/DNA-PKcs, thereby facilitating DNA-PKcs phosphorylation at S2056 and promoting efficient NHEJ (Chen et al., 2017). ATM-dependent phosphorylation of the deubiquitinase UCHL3 enables UCHL3 to enhance the retention of Ku80 at damage sites by counteracting Ku80 ubiquitination (Nishi et al., 2018). Notably, cAMP-mediated recruitment of XRCC4 and DNA ligase IV to DSB sites is PKA-dependent (Noh and Juhnn, 2020b). Finally, the DNA helicase RECQL4 promotes DNA-PKcs and Ku70/80-mediated DNA end bridging, as well as the accumulation/retention of NHEJ factors at DSBs (Lu et al., 2022).

Numerous other targets of DNA-PK exist. Upon activation, DNA-PK phosphorylates the N-terminal domain of Y-box binding protein YB-1 at T89, leading to its nuclear translocation (Nöthen et al., 2023), where it binds endonuclease III (hNTH1) and participates in DNA repair (Senarisoy et al., 2020). DNA-PK also promotes DNA repair by phosphorylating ZAK at T168 to activate the ZAK/AKT/mTOR pathway (Wang et al., 2024). Additionally, DNA-PK phosphorylates Sin1 within the mTOR complex 2 (mTORC2), facilitating its interaction with the guanine nucleotide exchange factor ECT2 to activate protein kinase B (AKT/PKB) and trigger the DNA damage response (DDR) (Liu L et al., 2022). DNA-PK can also phosphorylate p53, causing cell cycle arrest at the G1/S phase in a P21-dependent manner (Sesink et al., 2024). Moreover, DNA-PK suppresses HR repair in the G1 phase by upregulating the expression of RING-box protein 1, thereby promoting NHEJ (Xie et al., 2020). Additionally, the Ku70/80 heterodimer regulates ATM and ATR signaling pathways during DNA DSB repair, modulating the activity of ATM and other phosphatidylinositol (PI) 3-related kinases, as well as the phosphorylation of p53 (S18) during DSB formation (Tomimatsu et al., 2007).

## Key ATR signaling events under irradiation

Mechanistically, DNA-PK and ATM are primarily activated by DSBs, whereas ATR is activated by TopBP1 or ETAA1 on extended stretches of ssDNA covered with RPA (Bass et al., 2016), and during DSB, ATR activation is mainly promoted by end-resection at HR repair sites. Initially, RPA recognizes and coats ssDNA at the irradiation-induced DNA damage sites (MacDougall et al., 2007). Subsequently, upon RPA binding to ATR-interacting protein (ATRIP) and recruiting ATR, the ATR-ATRIP complex forms at the replication fork of DSBs (Zou and Elledge, 2003). The structure of the ATR-ATRIP complex differs from that of an ATM dimer, consisting of two conformationally different ATR monomers and two ATRIP molecules (Ball and Cortez, 2005). The formation of this complex is necessary for its activity (Ball and Cortez, 2005). Activation of the ATR-ATRIP complex also requires the participation of ATR activators such as TOPBP1 and ETAA1 (Kumagai et al., 2006; Bass TE et al., 2016). TOPBP1 activates the complex by interacting with ATR and ATRIP through its ATR activator domain (AAD) (Kumagai et al., 2006). ETAA1 contains two RPA interaction motifs, which localize ETAA1 to stalled replication forks and directly bind ATR/ATRIP using motifs similar to those in the TOPBP1 ATR activation domain (Bass et al., 2016). Depending on the cell cycle stages and the presence or absence of DNA damage, the ATR-ATRIP complex is activated by different ATR activators. In the presence of exogenous DNA damage during replication, ATR signaling mainly depends on TOPBP1 (Saldivar et al., 2018), while during normal DNA replication and mitosis, ETAA1 is the primary ATR activator (Bass et al., 2019).

When cells are exposed to IR, the DSBs repair pathways collaborate with DNA damage cell cycle checkpoints to maintain genomic stability. Studies have demonstrated a dose-dependent interaction between ATM, ATR, and DNA-PKcs in G2 phase cells exposed to varying doses of IR (Mladenov et al., 2019a). Under low IR dose conditions, there is a tight interconnection between homologous recombination repair (HRR) and the ATM/ATR-dependent G2 checkpoint, with HRR playing a predominant role; inhibiting either one can completely suppress resection (Soni et al., 2021). High IR doses generate more DSBs, which relaxes the tight coupling between ATM and ATR, leading to pronounced resection output (Xue et al., 2015). Under conditions of high Linear Energy Transfer (LET)-IR, increased DNA end resection inhibits c-NHEJ repair of DSBs, and elevated DNA end resection activates ATR, primarily regulating the G2 checkpoint (Fujisawa et al., 2015). DNA-PKcs can regulate resection under all doses of IR, integrating with the ATM/ATR module. The modular unit formed by these three factors demonstrates significant crosstalk in the regulation of DSB processing during the S and G2 phases (Mladenov et al., 2019b).

In the DDRs induced by IR, NHEJ is active throughout the cell cycle, repairing DSBs by directly connecting the DNA break ends (Averbeck et al., 2014). However, complex DNA cluster damage induced by high LET IR results in cells primarily using HR to repair these damages during the S and G2 phases, which involves resection of DSB ends before proceeding with repair (Nickoloff, 2021; Zhou et al., 2021). HR requires an early process of DNA end resection to generate ssDNA, initiated jointly by the MRE11 nuclease and CtIP at the DSB ends (Averbeck et al., 2014). This process first produces short ssDNA tails and then recruits downstream nucleases and helicases such as EXO1, DNA2, or BLM for extensive resection amplification (Nimonkar et al., 2011; Tomimatsu et al., 2012) to extend the 3′-ssDNA. During the end resection elongation process, RPA coats ssDNA and participates in the regulation of DNA2/BLM activity. When the length of ssDNA is sufficient for HR repair, recruited ATM/ATR targets the end resection regulatory proteins through different pathways to terminate resection. ATR-mediated phosphorylation of EXO1 promotes EXO1 degradation (Tomimatsu et al., 2017), while ATM-mediated phosphorylation of EXO1 regulates its activity after end resection, leading to RPA dissociation and the completion of HR repair (Bolderson et al., 2010). In the HR-mediated DDR, when blunt ends or short single-strand overhangs (SSO) are excised, the SSO reduces ATM activation and enhances ATR activation in a length-dependent manner. The progressive end resection of DSBs directly facilitates the switch from ATM to ATR activation (Shiotani and Zou, 2009).

The recruitment of ATR activators to stalled replication forks is closely related to ATM. When 5′-end ssDNA-dsDNA junctions are present, the RAD17 replication factor (Zou et al., 2003), or Ctf18-RFC and DNA Pol ε (Stokes et al., 2020), assist in loading RAD9-RAD1-HUS1 (9-1-1) checkpoint clamp complexes (Bermudez et al., 2003), which subsequently recruit TOPBP1 and stimulate ATR kinase activity (Delacroix et al., 2007). The recruitment of TOPBP1 to the 9-1-1 clamp also partially relies on the MRN complex (Duursma et al., 2013) and the RHINO protein (Cotta-Ramusino et al., 2011), although the specific mechanism remains unclear. While ETAA1 contains an ATR activation domain similar to TOPBP1, it has been reported that it can also interact with RPA (Haahr et al., 2016). Furthermore, studies have indicated that the dimerization of TOPBP1 and ETAA1 can enhance ATR signal transduction and promote the activation of ATR kinase (Thada and Cortez, 2021). It is also noteworthy that CIP2A interacts with TOPBP1, allowing DNA-damaged cells entering mitosis (Nagelli and Westermarck, 2024).

To a lesser extent, oxidative base damage caused by IR may contribute to replication stress and subsequent ATR activation. The main repair pathways for IR-induced oxidative DNA damage are base excision repair (BER) and nucleotide excision repair (NER) (Pawar et al., 2009; Popanda et al., 2013). Radiation sites induce the oxidation of bases such as 7, 8-dihydro-8-oxyguanine (8-oxoG) and thymine glycol (TG). When cells initiate the BER pathway, DNA glycosylases OGG1 and NTH1, which cleave 8-oxoG and TG respectively, are recruited to the damaged site (Robeska et al., 2024). Once the initial oxidative damaged base is cleaved by DNA glycosylase, apurinic/apyrimidinic (AP) sites are produced. AP endonucleases or AP lyases can create SSBs when cleaved at the AP site. The most typical AP endonucleases (APE) are APE1 and APE2 (Willis et al., 2013). In BER, the cleavage of the AP site produces ssDNA/dsDNA junctions, recruiting PCNA, followed by APE2 and CHK1. APE2 further cleaves to produce long segments of ssDNA, assembling checkpoint protein complexes including ATR, ATRIP, and the 9-1-1 complex onto RPA-bound ssDNA, which is then linked through TopBP1, leading to ATR activation. The activated ATR phosphorylates CHK1 and the 9-1-1 complex, acting as a positive feedback mechanism to stimulate the BER pathway (Willis et al., 2013).

The activation of ATR triggers the phosphorylation of various downstream targets, with CHK1 being one of the most critical. Activated ATR phosphorylates CHK1 at S345 and S317 (Wang et al., 2012), facilitating the degradation of CDC25A through the ubiquitin-proteasome pathway (Mailand et al., 2000). This degradation reduces CDK2 activity and halts cell cycle progression, providing time for DNA repair (Ditano et al., 2021). Additionally, activated CHK1 can induce the inactivation/degradation of Cdc25C, thereby inhibiting the transition from G2 to M phase by blocking dephosphorylation of CDK1 (55). CHK1 also contributes to cell cycle deceleration by activating WEE1 kinase, which phosphorylates CDK1 (Durinikova et al., 2022). The CHK1-mediated inactivation/degradation of CDC25A and CDC25C shares significant overlap with CHK2 downstream pathways in ATM. Moreover, CHK1 also promotes HR through the phosphorylation of BRCA1, BRCA2, and RAD51 (Fan et al., 2020; Peng et al., 2021) and promotes NHEJ through the phosphorylation of DNA-PK (Mladenov et al., 2019a). CHK1 and CHK2 can both regulate the cell cycle through p53, another intersection point between the ATM and ATR pathways (Song et al., 2015).

## Conclusion and discussion

DSBs induced by irradiation are severe lesions that can cause mutations, such as deletions or chromosomal translocations. NHEJ and HR are the two primary mechanisms for DSB repair, and cells tend to adopt the optimal repair pathway for a given situation. NHEJ can function during the G1/S/G2 phases, while HR is active only during the S and G2 phases of DNA replication (Mao et al., 2008). In S and G2 phase DSB repair, NHEJ accounts for approximately 70%, and HR accounts for approximately 30% (Shibata and Jeggo, 2020). In the NHEJ pathway for DSB repair induced by IR, 53BP1 is a key factor (Genetta et al., 2023). NHEJ directly ligates the broken DNA ends to repair DSBs (Averbeck et al., 2014). HR requires end resection of DSBs to generate 3′ssDNA, which can then recruit the recombinase RAD51 to repair the lesion using the sister chromatid as a template (Ira G et al., 2004).

53BP1 is a large chromatin-associated protein characterized by tandem BRCA1 C-terminal (BRCT) domains, a glycine/arginine-rich (GAR) domain, and a Tudor domain (Shibata and Jeggo, 2020). 53BP1 bind to chromatin minimally without DNA damage and is recruited to DSBs via interactions with nucleosomes containing H4K20me2 and damage-induced ubiquitinated H2AK15 (Fradet-Turcotte et al., 2013). Through ATM-dependent phosphorylation, 53BP1 recruits proteins such as RIF1 (Chapman et al., 2013), PTIP (Munoz et al., 2007), and the Shieldin complex (Sifri et al., 2023) to IR-induced DSB sites, thereby inhibiting resection and promoting NHEJ (Escribano-Díaz et al., 2013). The resection of DSBs requires BRCA1-dependent dephosphorylation of 53BP1 and the absence of RIF1 (Isono et al., 2017). BRCA1 is a large protein composed of 1,863 amino acids, containing an N-terminal RING domain and two C-terminal tandem BRCT domains. The BRCT domains recognize phosphoproteins with a phosphorylated serine-proline-x-phenylalanine (pSPxF) motif (Manke et al., 2003; Rodriguez et al., 2003), including ABRAXAS1, Bach1/FANCJ (BRIP1), and CtIP (Wu et al., 2016). IR-induced, ATM-dependent phosphorylation of S404 adjacent to the pSPxF motif stabilizes BRCT/ABRAXAS1 complex dimers, which regulates ABRAXAS1-mediated recruitment of BRCA1 (Wu et al., 2016). BRCA1 and cofactors such as CtIP, EXO1, BLM/DNA2, and the MRN complex promote DNA end resection, facilitating HR (Xu and Xu, 2020).

During the S and G2 phases of the cell cycle, CDK-mediated phosphorylation of FANCJ at S990 promotes its acetylation, and the recruitment of CtIP to DSB sites depends on the acetylation of FANCJ at K1249 (Nath and Nagaraju, 2020). Additionally, CDK phosphorylates CtIP at Thr847 and Ser327 (Yu and Chen, 2004; Huertas and Jackson, 2009), enabling its interaction with the BRCA1 and MRN complex, thereby maximizing homologous recombination efficiency (Buis et al., 2012). Under IR, low CDK activity affects CtIP activity, thereby altering the choice of DSB repair pathways (Li et al., 2023). Wee1-like protein kinase (WEE1) can inhibit CDK activity during the S and G2 phases, promoting the recruitment of 53BP1 (Petrosius et al., 2022). Therefore, the key for regulation is that BRCA1 reads out S/G2-CDK-mediated phosphorylation of ABRAXAS1, BRIP1 and CtIP via its BRCT domains, thereby initiating end resection, whereas 53BP1 counteracts BRCA1 recruitment in the absence of S/G2-CDK phosphorylation.

ATM is a key regulator of checkpoints during the G1, S, or G2 phases, while ATR plays a partially redundant role in regulating checkpoints during the S and G2 phases, and DNA-PKcs is not involved in checkpoint regulation (Li et al., 2021). With increasing radiation doses, the G1 checkpoint shifts from ATM-only to ATM-plus-ATR regulation (Li et al., 2021). DNA-PK is a core component of NHEJ, working with DNA damage checkpoint kinases ATM and ATR to influence HR repair of DSBs (Serrano et al., 2013). Novel responses of DNA damage have been emerging, for example, cancer cells have developed a strategy to keep alive under irradiation, by increasing DNA breaks mediated by caspase-activated DNase (CAD) whose activity is dependent on ATM/ATR/DNA-PK activities induced by DNA damage. The increase of the “self-inflicted” DNA breaks therefore prevents the defective cells from entering mitosis and collapse (Larsen et al., 2022), and suggests targeting G2 checkpoint for effective treatment of these cells. This review consolidates recent research on the molecular mechanisms underlying IR-induced DNA damage, and provides a comprehensive yet brief description of the key molecular events of the cellular responses to reflect the current understanding of the key mechanisms involved overall. It also provides important insights for studying radiotherapy resistance. In the future, studies of therapeutic effects as well as corresponding molecular mechanisms of various combined therapies involving irradiation will be important directions for cancer workers.
